# The Role of Interleukin-1 Genotype in the Association between Coronary Heart Disease and Periodontitis in a Syrian Population

**DOI:** 10.1155/2013/195678

**Published:** 2013-04-14

**Authors:** Lina Bashour, Razan Khattab, Elham Harfoush

**Affiliations:** ^1^Department of Periodontology, Faculty of Dentistry, Damascus University, Al-Mezzeh, Damascus, Syria; ^2^Department of Microbiology and Genetics, Faculty of Medicine, Damascus University, Damascus, Syria

## Abstract

*Objective*. To determine whether differences exist between periodontitis subjects with and without Coronary Heart Disease (CHD) in a Syrian population in the distribution of IL-1 alleles at positions IL-1*α*+4845, IL-1*β*+3954, IL-1*β*−511, and IL-1RN VNTR. *Background*. The role of Interleukin-1 genes in the association between periodontitis and CHD has been demonstrated in previous studies. No study has been carried out on the Syrian population to asses for such a role. *Methods*. 200 Syrian Arab periodontitis patients (184 males, 16 females; mean age 52.61) were divided into two groups: cases group 100 subjects with CHD (92 males, 8 females; mean age 52.06); controls group 100 subjects without CHD (92 males, 8 females; mean age 53.16). Probing depth (PD), clinical attachment loss (CAL), and alveolar bone loss (ABL) were performed for patients. Blood samples were collected for genotyping analysis of IL-1*α*+4845, IL-1*β*+3954, and IL-1*β*−511 using PCR-RFLP technique and IL-1RN VNTR using normal PCR. *Results*. An association between both (CAL and ABL) and CHD was shown after adjustment for other confounders (OR: 7.659, *P* = 0.001; OR: 3.645, *P* = 0.006, resp.). Also, an association between allele 2 of IL-1*α*+4845, IL-1*β*+3954, and IL-1*β*−511 and ABL was shown. Allele 2 of IL-1*α*+4845 and IL-1*β*−511 was associated with ABL among individuals with and without CHD. But after adjustment for other confounders, the association remained only between allele 2 of IL-11*α*+4845 and both CHD and severe ABL (OR: 0.189, *P* < 0.001). *Conclusion*. Allele 2 of IL-11*α*+4845 may be considered a risk indicator for having both CHD and severe ABL in the investigated Syrian population.

## 1. Introduction

Periodontitis is a chronic inflammatory disease of multifactorial etiology initiated by specific bacteria that activate host mechanisms which in turn destroy the bone and connective tissues that support the teeth [1]. In recent years, studies have demonstrated that periodontitis is associated with elevated levels of inflammatory cytokines [2], which have a substantial impact on numerous biological activities, and they take part in triggering inflammatory cascades and systems [3]. To illustrate, Interleukin-1 (IL-1) plays a prominent role in the inflammatory response in periodontal lesions. IL-1*α* and IL-1*β* upregulate prostaglandin E2 and matrix metalloproteinase and, together with these components, promote the loss of connective tissue and bone in periodontitis lesions [4].

Atherosclerosis is considered the most common cause of Coronary Heart Disease (CHD). It is a variable combination of changes of the intima of arteries that lead to the narrowing of the lumen of the coronary arteries [5]. There is now a convincing body of evidence that atherosclerosis is much more than the simple vascular accumulation of lipids and has a major inflammatory component [6]. For instance, IL-1 plays a prominent role in the inflammatory response in atherosclerosis, stimulates the vascular smooth muscle cells by transforming growth factor-beta [7] and the expression of adhesion molecules by endothelial cells, promoting coagulation and thrombosis [8], and induces the synthesis of C-reactive protein [9] and other inflammatory mediators involved in atherosclerotic plaque formation [10, 11]. 

Earlier epidemiologic studies showed an association between periodontitis and CHD. The findings of meta-analysis have concluded that periodontitis is a risk factor or marker independent of traditional CHD risk factors with relative risk estimates ranging from 1.24 to 1.35 [12, 13]. Consistent with these findings, an earlier study of our group found out a positive association between periodontitis and atherosclerosis (OR: 2.4 : 1, 95% CI: from 1.57 to 3.68, and *P* < 0.05) after adjusting for other risk factors [14]. The epidemiological association between periodontitis and CHD has led to the identification of the biological mechanisms that explain the association [15]. Some hypotheses havesuggestedin an attempt to explain the association including the direct involvement of periodontal bacteria in the atheroma processes [16], the direct involvement of inflammatory mediators from periodontitis on the atheroma processes [17, 18], common predisposing mechanisms that influence both diseases [17, 18], and shared genetic predisposition that influences both diseases [15]. Although there is limited data to support a specific candidate gene as the explanation for observed associations between the two diseases, a few candidates look promising. One candidate that influences inflammation, IL-1 gene polymorphisms, has been associated with periodontitis and CHD [15]. IL-1 family genes are located in a cluster on human chromosome 2q13; a specific genotype in the IL-1 cluster that includes a specific locus is associated with increased IL-1 production [19]. 

Previously, variations in the genes that regulate the IL-1 response have been associated with periodontitis; allele 2 of IL-1*α*−889 and IL-1*β*+3954 was found to be associated with severe chronic periodontitis in nonsmokers [20, 21]. The composite genotype was found to be significantly associated with severe chronic periodontitis [22, 23]. A review and meta-analysis showed that IL-1*α* and IL-1*β* genetic variations are significant contributors to chronic periodontitis in Caucasians [24].

In addition, variations in the genes that regulate the IL-1 response have been associated with CHD; allele 2 of IL-1RN VNTR*2 (IL-1RN*2) was significantly associated with single vessel disease (SVD) [25] and carotid atherosclerosis [26]. The presence of an IL-1RN*2 polymorphism, known to increase IL-1Ra levels and possibly the IL-1Ra: IL-1 beta ratio, was associated with reduced coronary atherosclerosis [27]. A review found that IL-1 gene variations that are associated with over expression of inflammatory mediators correlate also with increased risk of cardiovascular events [28].

Moreover, several studies reported an association between IL-1 polymorphisms and periodontitis as well as CHD; one pattern of IL-1 genetic polymorphisms, characterized by the IL-1*α*+4845 and IL-1*β*+3954 markers, is associated with periodontitis. Another IL-1 genetic pattern, characterized by the IL-1*β*−511 and IL-1RN+2018 markers, is associated with atherosclerotic plaque formation [29]. IL-1 gene polymorphisms were found to play a role in the development of coronary artery disease (CAD), especially myocardial infarction (MI), in patients with *chlamydia pneumoniae *infection [30]. Patients with acute coronary syndrome (ACS) or angina were more likely to evidence a positive IL-1 polymorphism and severe periodontitis [31]. Allele 1 of IL-1RN VNTR was associated with the coexistence of CHD and periodontitis in a multiple regression model [32]. Modifying impact of the composite IL-1 genotype failed to be detected even though an association between periodontitis and acute myocardial infarction (AMI) was confirmed [33]. 

Indeed, the role of Interleukin-1 genes in the association between periodontitis and CHD has been demonstrated in previous studies. Thus, since no previous study has tested this role on a Syrian population the current study aimed to investigate the association between the polymorphism of the IL-1 gene cluster with periodontitis and CHD in the investigated Syrian population. 

## 2. Material and Methods

The study protocol was approved by the Research Ethics Committee of the Faculty of Dentistry, Damascus University, Damascus, Syria. Informed written consent was obtained from participants. 

The study was conducted as a matched case-control study from 2010 to 2012.

### 2.1. Study Population

The sample consisted of 200 Syrian Arab patients with periodontitis, (184 males, 16 females; aged 40–70 years, mean age 52.61) who were divided into two groups: the cases group consisted of 100 subjects with CHD (92 males, 8 females; mean age 52.06) from the Cardiosurgery Department of Al-Assad University Hospital, Damascus University; and the controls group consisted of 100 subjects without CHD (92 males, 8 females; mean age 53.16) who were matched for gender and age within two years. They were enrolled from the Department of Periodontology, Faculty of Dentistry, Damascus University. Criteria for exclusion from the study were the presence of diabetes mellitus, hypertension, high cholesterol levels, osteoporosis, pregnancy, and pronounced obesity (body mass index (BMI) > 30 kg/m^2^). Patients who presented <6 teeth were also excluded from the study. All patients were required to have Syrian Arab parents and grandparents to reduce genetic heterogeneity in the sample. 

### 2.2. Medical Examination

All participants had a medical examination, including a questionnaire on CHD and periodontitis risk factors. Hospital case records were reviewed to confirm the diagnosis of individuals with CHD. Diagnosis of CHD was based on coronary artery angiography. The cases group patients admitted to hospital were subject to coronary artery bypass graft, where the controls patients were referred to a specialist in cardiology to conduct an electrocardiogram to determine the absence of CHD.

### 2.3. Oral Examination

The same dentist performed all oral examinations and analyses of radiographs. Diagnosis of periodontitis was based on the existence of one side or moreprobing depths (PDs)≥ 4 mm or clinical attachment loss (CAL) ≥ 3 mm [35]. 

PD and CAL were performed for every patient using UNC dental probe. PD was measured as the distance from the gingival margin to the bottom of the pocket at four sites per tooth. CAL (distance between the cement-enamel junction and the bottom of the pocket) was obtained by adding the PD values to the gingival recession (GR) values (distance between the gingival margin and the cement-enamel junction) [36].

Alveolar bone loss (ABL) was measured using apical digital radiography. ABL was defined as the distance between the cement-enamel junction and the most apical level of the alveolar crest. ABL was stratified into three groups according to the American Dental Association (ADA) classification: ADAII group mean was 3-4 mm, ADAIII group mean was 4–6 mm, and ADAIV group mean was ≥6 mm [37].

### 2.4. Purification of DNA and Analyses of Genetic Polymorphisms

DNA extraction and genotyping IL-1 tests were achieved in Genetic Research Laboratory, Faculty of Medicine, Damascus University, Damascus, Syria. Venous blood samples were collected from the patients in vacutainer tubes containing EDTA. The blood samples were stored at −20°C until genomic DNA was isolated using Genomic DNA purification with NucleoSpin Blood, Macherey-Nagel (MN) kit, Germany. 

Genotypes of IL-1*α*+4845, IL-1*β*+3954, and IL-1*β*−511 were analyzed by Restriction Fragment Length Polymorphism (RFLP). Genotypes of the IL-1RN VNTR polymorphism were detected by normal Polymerase Chain Reaction (PCR) amplification and fragment size analysis. IL-1*α*+4845 is a nonsynonymous, biallelic polymorphism located on exon 5 of the IL-1a gene. IL-1*β*+3954 is a biallelic, synonymous polymorphism located on exon 5 of the IL-1*β* gene. IL-1*β*−511 is a biallelic promoter polymorphism of the IL-1*β* gene [19], and IL-1RN VNTR is a variable number of tandem repeats polymorphism located on intron 2 of the IL-1Ra gene [38].

PCR reactions consisted of 2.5 *μ*L DNA, 12.5 *μ*L PCR master mix (PCR Master Mix (2X), Fermentas Life Sciences, Canada), 9 *μ*L water for PCR, and 0.5 *μ*L each primer (VBC Biotech, Austria) (Table 1) in a final volume of 25 *μ*L. The PCR amplification conditions consisted of 95°C for 2 minutes, followed by 35 cycles of 95°C for 60 seconds and polymorphism-specific annealing temperature (Table 1) for 60 seconds and 72°C for 60 seconds. The PCR was terminated by final elongation at 72°C for 5 minutes. PCR amplification was performed in a thermal cycler ( TECHNE-TC-512 thermal cycler, UK). The amplified DNA was digested with restriction enzymes ( Fermentas Life Sciences, Canada) as listed in Table 1. Agarose gel electrophoresis and ethidium bromide staining analyzed all PCR products and digested PCR products. Genotypes were determined by comparing the size of the bands with a 100-base pair DNA ladder. The resulting products are listed in Table 1.

### 2.5. Statistical Analysis

Statistical analysis was performed using statistical software SPSS program V. 10 for Windows, Chicago, IL, USA, with *P* value < 0.05 considered statistically significant. 

The Mann-Whitney *U* test was used to assess differences of periodontal parameters between the two groups. 

Using the Pearson chi-square test, the Hardy-Weinberg equilibrium (HWE) was tested for fitness for IL-1 gene polymorphisms at positions (*α*+4845, *β*+3954, *β*−511, and RN VNTR). The chi square testes were utilized to detect any association between ABL, CHD, and IL-1 genotypes or alleles at positions (*α*+4845, *β*+3954, *β*−511, and RN VNTR). Multiple logistic regression was used to determine the role of IL-1 polymorphism at positions (*α*+4845, *β*+3954, *β*−511, and RN VNTR) in CHD, while adjusting for potential confounders. Odds ratio (OR) was calculated with 95% confidence interval (CI). 

## 3. Results

### 3.1. Descriptive Data and Periodontal Examination

Two hundred Syrian Arab patients with periodontitis (184 males, 16 females; mean age 52.61) were enrolled in the study. Subject characteristics are presented in Table 2. 

There were no relevant differences between cases and controls for smoking (*P* = 0.757, Table 2). The group of patients with CHD had significantly higher BMI values than the controls group (*P* = 0.005, Table 2). 

For all periodontal parameters assessed, cases showed worse results than controls. But CAL values were statistically, significantly higher among CHD patients (*P *< 0.001, Table 2). 

There was a statistically significant difference in the occurrence of ABL between the CHD group and non-CHD group (*P* = 0.016); the CHD group was more likely to evidence severe ABL compared to controls group (52% versus 32%, resp., Table 2).

### 3.2. IL-1 Allele and Genotype Frequencies

None of the polymorphisms genotyped deviated significantly from the assumption of the Hardy-Weinberg equilibrium (Table 1).

Table 3 shows groups of various IL-1 genotypes induced by CHD. There were significant differences in allele 2 of IL-1*α*+4845 between the CHD group and non-CHD group, while there were no significant differences in allele 2 of IL-1*β*+3954, IL-1*β*−511, and IL-1 RN VNTR between the CHD group and non-CHD group.

Table 4 shows groups of various IL-1 genotypes induced by ABL. There were statistically significant differences in allele 2 of IL-1*α*+4845, IL-1*β*+3954, and IL-1*β*−511 between ABL groups, while there was no statistically significant difference in allele 2 of IL-1 RN VNTR between ABL groups.

Table 5 shows IL-1 genotypes induced by ABL in individuals with and without CHD; allele 2 of IL-1*α*+4845 and allele 2 of IL-1*β*−511 were associated with ABL among individuals with and without CHD, while allele 2 of IL-1*β*+3954 and allele 2 of IL-1 RN VNTR were not associated with ABL among individuals with and without CHD.

The multiple logistic regression analysis showed a significant association between allele 2 of IL-1*α*+4845 and CHD when polymorphism of IL-1*α*+4845, IL-1*β*+4845, IL-*β*−511, and IL-1 RN VNTR, BMI, smoking, PD, GR, CAL, and ABL were included in a multiple logistic regression model (Table 6).

In a multiple logistic regression model, allele 2 of IL-1*α*+4845 was found to be associated with the occurrence of both CHD and (moderate + severe) ABL. This association was significant after an adjustment for polymorphism of IL-1*α*+4845, IL-1*β*+3954, IL-1*β*−511, and IL-1 RN VNTR and smoking, PD, GR, CAL, and BMI (Table 7).

## 4. Discussion

The comparability between cases and controls is critical in case-control studies of polymorphisms. Because periodontitis and CHD share common risk factors, it is important that the controls have the same burden of risk factors exposure as do the cases. That is why the non-CHD group was matched for gender and age with the CHD group. Subjects with diabetes mellitus, blood hypertension, hyperlipidemia, and BMI > 30 kg/m^2^ were excluded from the study population. There was no relevant difference between cases and controls for smoking (*P* > 0.05). However, because patients with CHD were examined before controls and most of them were males 92%, this resulted in a small number of females in the study population 8%.

Previous studies used different clinical criteria for periodontitis. In our study, periodontal pathology was assessed through PD, CAL, and ABL, while hygiene indexes were not included because patients hospitalized after coronary artery bypass graft could be expected to neglect their oral hygiene and, therefore, to have worse indexes. Additionally, the majority of patients with CHD received anticoagulant drugs, which increase gingival bleeding and, thus, raise doubt about the value of using hygiene indexes as diagnostic criteria in our study. To further consider the extent and severity of periodontitis, we differentiated mild, moderate, and severe forms of periodontitis based on ADA classification of ABL [37], because radiographic evidence of alveolar bone loss was considered the best individual parameter for associating periodontitis with AMI [39]. 

The results of our clinical investigation demonstrated a worse periodontal health status in patients with CHD compared to controls. CAL values were statistically, significantly higher among CHD patients (*P *< 0.001). After adjustment for smoking and other confounders, the association between CAL and CHD remained significant (OR: 3.645, 95% CI: 1.462–9.083, and *P* = 0.006) (Table 7), which confirms the previous findings [33]. There was also a strong association between ABL and CHD after adjustment for smoking and other confounders (Table 6). The OR (severe + moderate compared to controls) was 7.659 (95% CI: from 2.357 to 24.886,* P* = 0.001). Thus, our results suggest that ABL might be a risk indicator for CHD and, thereby, confirm the findings of the majority of previous studies [32, 39, 40]. 

In this study, we found out an association between allele 2 of IL-1*α*+4845 and CHD (OR: 3.03, 95% CI: from 1.70 to 5.40, and *P* < 0.001). The OR remained significant also when other risk factors were excluded (OR: 0, 189, 95% CI: from 0.039 to 0.383, and *P* < 0.001). Therefore, our results suggest that allele 2 of IL-1*α*+4845 might be a risk indicator for CHD, and thereby these findings were in agreement with previous studies indicating that patients with genotype 1.2 or 2.2 at the IL-1*α*+4845 polymorphism had higher median CRP (2.92 versus 2.05 mg/L,* P*i = 0.023), and CRP levels remained significantly associated with IL-1 polymorphisms after adjustment for smoking, gender, and age [41].

We did not find an association between allele 2 of IL-1*β*+3954 and CHD, which confirms the findings of several previous studies [32, 33, 42]. In addition, no association between allele 2 of IL-1*β*-511 and CHD was found, which is in agreement with several previous studies [32, 42, 43]. This result was different from those reporting earlier that −511C/T IL-1 beta gene polymorphism affects the risk of MI and ischemic stroke at young age and the response of mononuclear cells to inflammatory stimulation [44]. Even though we found a higher frequency of allele 2 of IL-1 RN VNTR in the CHD group compared to non-CHD group (46% versus 57%, resp.), it remained insignificant. This was in agreement with several studies [32, 42, 43] and in contrast to others [25, 26, 45, 46].

In addition, in this study, we found an association between allele 2 of IL-1*α*+4845, IL-1*β*+3954, and IL-1*β*−511 and ABL (*P* < 0.05, Table 4). These findings are in contrast to several previous studies [20, 47, 48] and in agreement with others [32, 49–51] indicating that IL1*α* and IL1*β* genetic variations are significant contributors to chronic periodontitis in Caucasians [24]. Allele 2 of IL-1*β*+3954 is associated with a 4-fold increase in IL-1*β* production [52]. IL1*α* and IL-1*β* production upregulates prostaglandin E2 and matrix metalloproteinase and, together with these components, promote the loss of connective tissue and bone in periodontitis lesions [4]. 

Moreover, in this study, polymorphisms of IL-1*α*+4845 and IL-1*β*−511 were associated with ABL among individuals with and without CHD (*P* < 0.05), while polymorphisms of IL-1*β*+3954 and IL-1 RN VNTR were not associated with ABL among individuals with and without CHD (*P* > 0.05). After adjustment for smoking and other confounders, only the association between allele 2 of IL-1*α*+4845 and the occurrence of both CHD and (moderate + severe) ABL remained significant. Thus, our results suggest that allele 2 of IL-1*α*+4845 might be a risk indicator for CHD and severe periodontitis. While these results do not support that allele 2 of IL-1 RN VNTR may explain the coexistence of periodontitis and CHD, they confirm the previously reported findings that the SNP at IL-1*α*−889 has been associated with different levels of IL-1*α* in human gingival tissue fluid and the SNP at IL-1*α*+4845 codes for an altered amino acid sequence in the IL-1*α* protein [53]. In addition, the individuals who carried two copies of IL-1*α*+4845 allele 2 were four times more likely to have a CHD event during an 11-year monitoring period than were individuals with the same level of cholesterol but who did not carry this IL-1 genotype [54].

Indeed, the varying results between our findings and previous findings could be due to, first, the ethnic variation in the population samples under investigation, because the frequencies of polymorphisms are known to vary by ethnicity [55]. Second, the variables used to describe periodontitis or CHD differ from study to study. Third, the modifiable effect from environmental agents, including smoking, could differ between phenotypes. Forth, the relatively small sample sizes, which is a significant problem for most studies on the association between polymorphisms of IL-1 and periodontitis [33, 48, 49, 51], limit robust conclusions.

Therefore, future work, with larger sample size, to investigate the role of IL-1 haplotype in the association between CHD and periodontitis is required to provide further understanding of the association between periodontitis and CHD. 

## Figures and Tables

**Table 1:** Genotyping of the four polymorphic variants.

Gene/SNP	Primer sequences	Ta (°C)	RE	DT (°C)	DD (h)	PCR product (bp)	HWE *P* value
IL-1*α* +4845 rs17561 G>T	FW: 5′-ATGGTTTTAGAAATCATCAAGCCTAGGGCA-3′ Rev: 5′ATGAAAGGAGGGGAGGATGACAGAAATGT-3′	56	SatI	37	16	153	0.3124
IL-1*β* +3954 rs1143634 C>T	FW: 5′-CTCAGGTGTCCTCCAAGAAATCAAA-3′ Rev: 5′-GCTTTTTTGCTGTGAGTCCCG-3′	57	TaqI	65	2	182	0.0591
IL-1*β* −511 rs16944 A>G	FW: 5′-TGGCATTGATCTGGTTCATC-3′ Rev: 5′-GTTTAGGAATCTTCCCACTT-3′	55	AvaI	37	16	304	0.547
IL-1 RN VNTR	FW: 5′-CTCAGCAACACTCCTAT-3′ Rev: 5′-TCCTGGTCTGCAGGTAA-3′	55				410 = 4 repeats 240 = 2 repeats	0.562

SNP: single nucleotide polymorphisms; Ta: annealing temperature; HWE: the Hardy-Weinberg equilibrium; RE: restriction enzyme; DT: digestion temperature; DD: digestion duration; rs numbers are RefSNP SNP-identification codes as applied in the public nucleic acid polymorphism databases at the national center for biotechnology information [34].

**Table 2:** Descriptive parameters and periodontal parameters in patients with and without CHD.

Variable	CHD	Non-CHD	

	*n* = 100	*n* = 100	*P* value

Age (years; mean ± SD)	52.06 ± 6.899	53.16 ± 5.832	0.225^†^
Male (%)	92%	92%	1.00^†^
Smokers (%)	68%	63%	0.757^†^
BMI (Kg/m^2^; mean ± SD)	26.17 ± 2.03	25.87 ± 1.91	0.005^†^

			*U *value

PD (rank)	106.11	94.90	0.158^‡^
GR (rank)	105.48	95.53	0.223^‡^
CAL (rank)	121.07	79.93	<0.001^‡^
ABL			
Mild (%)	8%	12%	0.016*
Moderate (%)	40%	56%	
Severe (%)	52%	32%	

^†^Unpaired *t*-test.

^‡^Mann-Whitney *U* test

*X ^2^ test.

**Table 3:** Genotype frequency of polymorphisms in the IL-1 gene cluster by CHD and Non-CHD.

Genotype frequency	CHD		Non-CHD		*P* value
	*n*	%	*n*	%
IL-1*α* +4845					
1.1	64	64%	37	37%	<0.001
Combination 1.2 + 2.2	36	36%	63	63%	
IL-1*β* +3954					
1.1	55	55%	52	52%	0.671
Combination 1.2 + 2.2	45	45%	48	48%	
IL-1*β* −511					
1.1	43	43%	40	40%	0.667
Combination 1.2 + 2.2	57	57%	60	60%	
IL-1 RA VNTR					
1.1	46	46%	57	57%	0.120
Combination 1.2 + 2.2	54	54%	43	43%	

**Table 4:** Genotype frequency of polymorphisms in the IL-1 gene cluster by the varying degrees of ABL.

	Alveolar bone loss						
Genotype frequency	Mild		Moderate		Severe		*P* value
	*n*	%	*n*	%	*n*	%	
IL-1*α* +4845							
1.1	19	95%	49	51%	33	39%	<0.001
Combination 1.2 + 2.2	1	5%	47	49%	51	61%	
IL-1*β* +3954							
1.1	14	70%	57	59%	36	43%	0.025
Combination 1.2 + 2.2	6	30%	39	41%	48	57%	
IL-1*β* −511							
1.1	9	45%	25	26%	49	58%	<0.001
Combination 1.2 + 2.2	11	55%	71	74%	35	42%	
IL-1 RA VNTR							
1.1	9	45%	44	46%	50	60%	0.154
Combination 1.2 + 2.2	11	55%	52	54%	34	40%	

**Table 5:** IL-1 genotypes of subjects with and without CHD by varying degrees of ABL.

	Alveolar bone loss						
Genotype frequency	Mild		Moderate		Severe		*P* value
	*n*	%	*n*	%	*n*	%	
IL-1*α* +4845							
CHD							
1.1	7	88%	31	78%	26	50%	0.009
Combination 1.2 + 2.2	1	12%	9	22%	26	50%	
Non-CHD							
1.1	12	100%	18	32%	7	22%	<0.001
Combination 1.2 + 2.2	0	0%	38	68%	25	78%	
IL-1*β* +3954							
CHD							
1.1	5	63%	27	68%	23	44%	0.070
Combination 1.2 + 2.2	3	37%	13	32%	29	56%	
Non-CHD							
1.1	9	75%	30	54%	13	41%	0.133
Combination 1.2 + 2.2	3	25%	26	46%	19	59%	
IL-1*β* −511							
CHD							
1.1	5	63%	9	23%	29	56%	0.003
Combination 1.2 + 2.2	3	37%	31	77%	23	44%	
Non-CHD							
1.1	8	33%	32	29%	40	63%	<0.001
Combination 1.2 + 2.2	16	67%	80	71%	24	37%	
IL-1 RA VNTR							
CHD							
1.1	5	63%	14	35%	27	52%	0.107
Combination 1.2 + 2.2	3	37%	26	65%	25	48%	
Non-CHD							
1.1	4	33%	30	54%	23	72%	0.054
Combination 1.2 + 2.2	8	67%	26	46%	9	28%	

**Table 6:** Multiple logistic regression with backward stepwise elimination.

Significant variable	*P* value
BMI	0.002
ABL (moderate + severe)	0.001
Allele 2 of IL-1*α* +4845	<0.001

Outcome variable is CHD.

Full model was allele 2 of IL-1*α* +4845, allele 2 of IL-1*β* +3954, allele 2 of IL-1*β*  −511, allele 2 of IL-1 RN VNTR, age, gender, smoking, BMI, PD, GR, CAL, and ABL.

**Table 7:** Multiple logistic regression with backward stepwise elimination.

Significant variable	*P* value
Age	0.015
BMI	<0.001
CAL	0.006
Allele 2 of IL-1a+4845	<0.001

Outcome variable is CHD and (moderate + severe) ABL.

Full model was allele 2 of IL-1*α* +4845, allele 2 of IL-1*β* +3954, allele 2 of IL-1*β*  −511, allele 2 of IL-1 RN VNTR, age, gender, smoking, BMI, PD, GR, and CAL.
